# Phospholipase Cγ1 links inflammation and tumorigenesis in colitis-associated cancer

**DOI:** 10.18632/oncotarget.23430

**Published:** 2017-12-19

**Authors:** Kwang-Il Park, Kwang-Youn Kim, Tae Woo Oh, Du-Seock Kang, Eung-Kyun Kim, Yong Ryoul Yang, Young-Kyo Seo, Jin-Yeul Ma, Pann-Ghill Suh

**Affiliations:** ^1^ Korean Medicine (KM)-Application Center, Korea Institute of Oriental Medicine (KIOM), Cheomdan-ro 70, Dong-gu, Daegu 41062, Republic of Korea; ^2^ School of Life Sciences, Ulsan National Institute of Science and Technology (UNIST), UNIST-gil 50, Ulsan 44919, Republic of Korea; ^3^ Gene and Cell Therapy Research Center for Vessel-associated Diseases, Pusan National University, Yangsan 50612, Republic of Korea

**Keywords:** phospholipase C γ1, inflammation, colorectal cancer, intestinal barrier dysfunction, inflammatory bowel disease

## Abstract

Colorectal cancer (CRC) is the third diagnosed cancer and the second leading cause of cancer-related deaths in the United States. Colorectal cancer is linked to inflammation and phospholipase Cγ1 (PLCγ1) is associated with tumorigenesis and the development of colorectal cancer; however, evidence of mechanisms connecting them remains unclear. The tight junctions (TJ), as intercellular junctional complexes, have an important role for integrity of the epithelial barrier to regulate the cellular permeability. Here we found that PLCγ1 regulated colitis and tumorigenesis in intestinal epithelial cells (IEC). To induce the colitis-associated cancer (CAC), we used the AOM/DSS model. Mice were sacrificed at 100 days (DSS three cycles) and 120 days (DSS one cycle). In a CAC model, we showed that the deletion of PLCγ1 in IEC decreased the incidence of tumors by enhancing apoptosis and inhibiting proliferation during tumor development. Accordingly, the deletion of PLCγ1 in IEC reduced colitis-induced epithelial inflammation via inhibition of pro-inflammatory cytokines and mediators. The PLCγ1 pathway in IEC accelerated colitis-induced epithelial damage via regulation of TJ proteins. Conclusions: Our findings suggest that PLCγ1 is a critical regulator of colitis and colorectal cancer and could further help in the development of therapy for colitis-associated cancer.

## INTRODUCTION

Cancer comprises a large family of diseases that is characterized by abnormal cell growth and invasion of surrounding tissues. Cancer cells undergo modified cellular processes, such as cell proliferation, migration, and differentiation, using altered signaling pathways. Colorectal cancer (CRC) is the third most frequently diagnosed cancer and the second leading cause of cancer-related deaths in the United States. Various mutations have been associated with the development of CRC, including the Wnt signaling pathway, K-Ras, p53, and the transforming growth factor β pathway [1, 2]. Cancer-associated inflammation, as a hallmark of cancer, is an important factor in the development of CRC; other environmental factors, such as epigenetic abnormalities and genetic mutations also play important roles in this process [3–5]. It has been reported that chronic inflammation is the leading cause of increased risk of several types of cancers including colon, liver, prostate, and breast cancer [6, 7]. Patients with inflammatory bowel disease (IBD), such as Crohn’s disease and ulcerative colitis, have a higher risk of developing CRC compared with healthy people. IBD, which is characterized by chronic inflammatory conditions of the gastrointestinal (GI) tract, results not only in intestinal epithelial barrier disruption but also in an influx of immune cells [8–10]. Tight junctions (TJs) of the intestinal epithelium play a pivotal role in the regulation of barrier function and protect the body from toxins, allergens, and pathogens in the intestine. IBD has been associated with an increased risk of CRC and the development of CRC between the stages of inflammation and carcinogenesis. This link between inflammation and the development of CRC has been extensively studied using mouse models of defects in the epithelial barrier in colitis and the formation of colorectal tumor. However, the contributing factors and underlying mechanisms have yet to be identified.

Phosphoinositide-specific phospholipase C (PLC) regulates cellular ligand-mediated signal transduction. PLC hydrolyzes phosphatidylinositol-4,5-bisphosphate (PIP2) into inositol-1,4, 5-triphosphate (IP3) and diacylglycerol (DAG). PLCs are activated by various extracellular ligands, such as growth factors, hormones, cytokines, and lipids. Several studies have shown that the activation of PLCs is associated with tumorigenesis and/or the progression of metastasis, such as migration, proliferation, growth, inflammation, angiogenesis, and the rearrangement of the actin cytoskeleton. As one of 13 mammalian PLC isozymes, PLC gamma 1 (PLCγ1) is primarily activated by extracellular stimuli and is involved in tyrosine kinase signaling and the promotion of tumor cell growth and migration [11, 12]. There is also evidence that PLCγ1 plays a critical role in cell adhesion and is highly expressed in metastatic tumors [13]. A genetic deficiency in PLCγ1 was shown to play an important role in metastasis and anti-apoptosis of human CRC, indicating that PLCγ1 can function as a oncogene *in vitro* [14].

Despite the increasing number of studies seeking to identify the possible link between PLCγ1 and tumorigenesis, the pathogenic role of PLCγ1 in inflammation-related cancers, particularly colitis-associated cancer (CAC), has yet to be investigated. The present study is the first to use intestine-specific PLCγ1 conditional knockout mice as an experimental model to investigate the role of PLCγ1 in intestinal inflammation and CAC. Ablation of PLCγ1 in intestinal epithelial cells (IECs) significantly ameliorated CAC progression. Our study sought to identify the role of PLCγ1 in tumorigenesis under physiological conditions.

## RESULTS

### Deletion of PLCγ1 in IECs decreases the incidence of CAC

**Figure 1:**
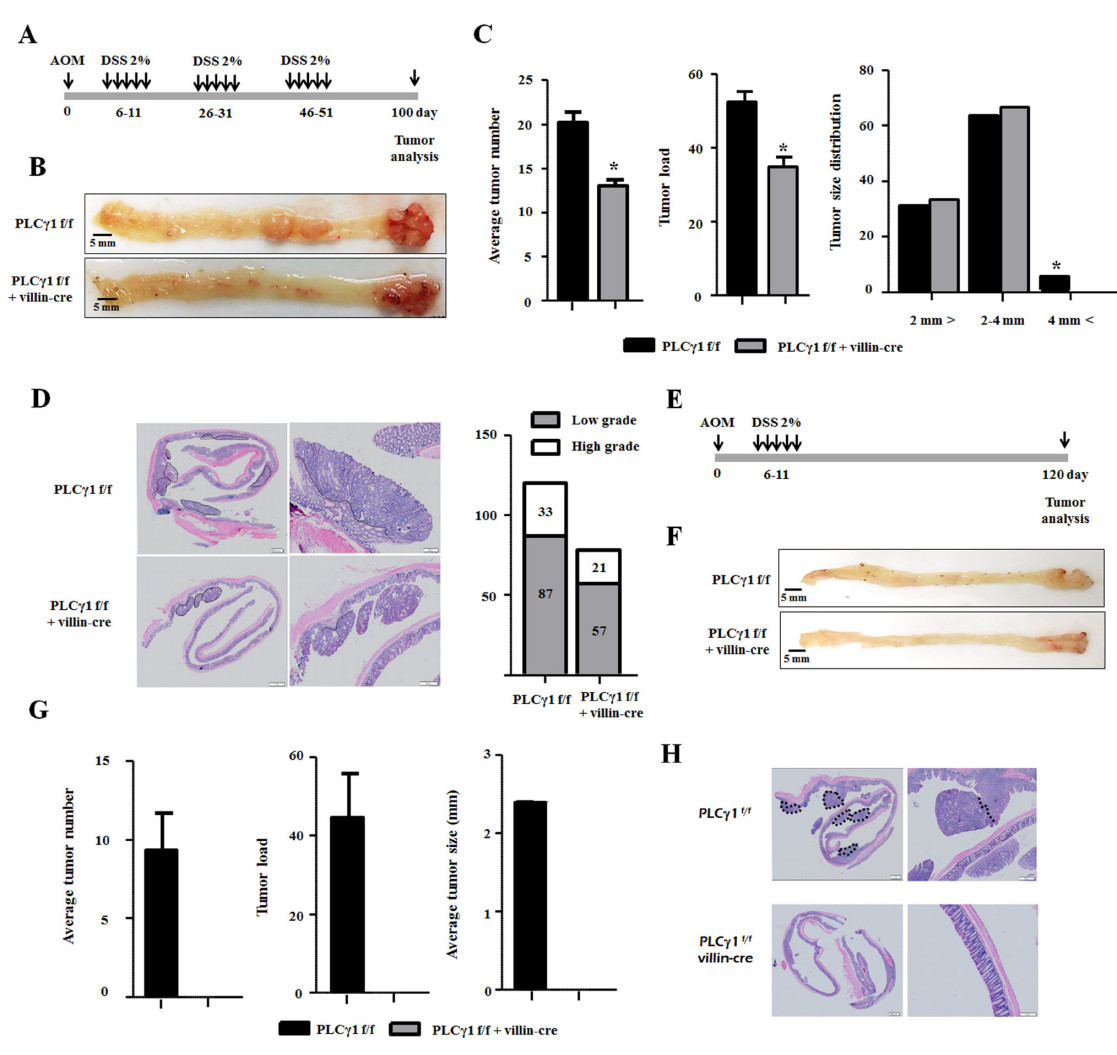
Deletion of PLCγ1 in IECs decreases AOM/DSS-induced tumor incidence in colorectal ducts (**A**) Design of the AOM/DSS protocol (DSS three cycles). (**B**) Representative images of colon tumors. (**C**) Average tumor number, load, and size distribution. Data represent the means ± SEM (*n* ≥ 6). ^*^p < 0.05. (**D**) H&E staining of tumors (**E**) Schematic representation of the AOM/DSS protocol (DSS one cycle). (**F**) Representative images of colon tumors. (**G**) Average tumor number, load, and size distribution. Data represent the means ± SEM (*n* ≥ 6). (**H**) H&E staining of tumor morphology.

Azoxymethane (AOM) acts as a carcinogen via the formation of O_6_-methyl guanine [15]. AOM/dextran sulfate sodium salt (DSS) was shown to induce tumors in the colons of rodent (distal to middle segments) and is commonly used in experimental CRC animal models [16]. We generated PLCγ1 IEC-specific knockout mice using Villin-Cre and PLCγ1 alleles to investigate the effects of the PLCγ1 deletion on IECs. We designed two experimental protocols to test the effects of inflammation on CAC. In the first study, 6–8 week-old mice were intraperitoneally (IP) injected with a single 10 mg/kg-dose of AOM followed by one or three cycles of 2% DSS administered in the drinking water (Figure 1A and 1E). Repeated DSS administration, used to mimic IBD, was conducted to cause AOM-induced tumors [17]. PLCγ1 conditional knockout mice and PLCγ1^f/f^ [wild type (WT)] littermates developed colon tumors, primarily in the distal to middle segments, using the AOM/DSS protocol (Figure 1B, 1D, 1F, 1G and 1H), consistent with the localization of human colorectal tumors, the most severe consequence of DSS-induced colitis [18]. However, mice receiving DSS alone did not produce tumors during the experimental period (data not shown). When PLCγ1 conditional knockout mice were subjected to three cycles of DSS, we observed a 50% decrease in the incidence of tumors, and the average tumor load was lower than that in WT mice. In addition, macroscopic tumors (>4 mm) were detected only in WT mice (Figure 1C). Histological analyses showed more low- and high grade tumors in WT mice than in PLCγ1 conditional knockout mice, but the relative proportion of low- versus high grade tumors was similar in both mouse groups. When mice were subjected to only one cycle of DSS (Figure 1E), we noticed that WT mice only manifested colon tumors, whereas no colon tumors were detected in PLCγ1 conditional knockout mice (Figure 1F and 1H). In addition, the incidence of tumors in WT mice undergoing one cycle of DSS was reduced by approximately 50% compared with WT mice undergoing three cycles of DSS.

This result suggests that the difference between the incidence of tumors causing an inflammatory response and the frequency of DSS administration is due to the effects of PLCγ1 on inflammatory responses caused by DSS and on both tumor initiation and development. Adenomatous polyposis coli (APC) or β-catenin gene mutations lead to increased tumor incidence through the stabilization of β-catenin and transcriptional activation with TCF-4, which play a pivotal role in CRC [19]. Genomic DNA from the tissue was isolated using laser capture microdissection and purified; exon 3 of the β-catenin gene was sequenced to look for mutations. We found that exon 3 of the β-catenin gene, which corresponds to a GSK3β phosphorylation sites, contained a serine-to-cysteine mutation in codon 33 in both WT and PLCγ1 conditional knockout mice (Supplementary Figure 1). These results suggested that the severity of inflammation is associated with increased tumor incidences and that PLCγ1 plays a pivotal role in tumor initiation and/or promotion.

### Deletion of PLCγ1 in IECs reduces proliferation and induces apoptosis in CAC

**Figure 2:**
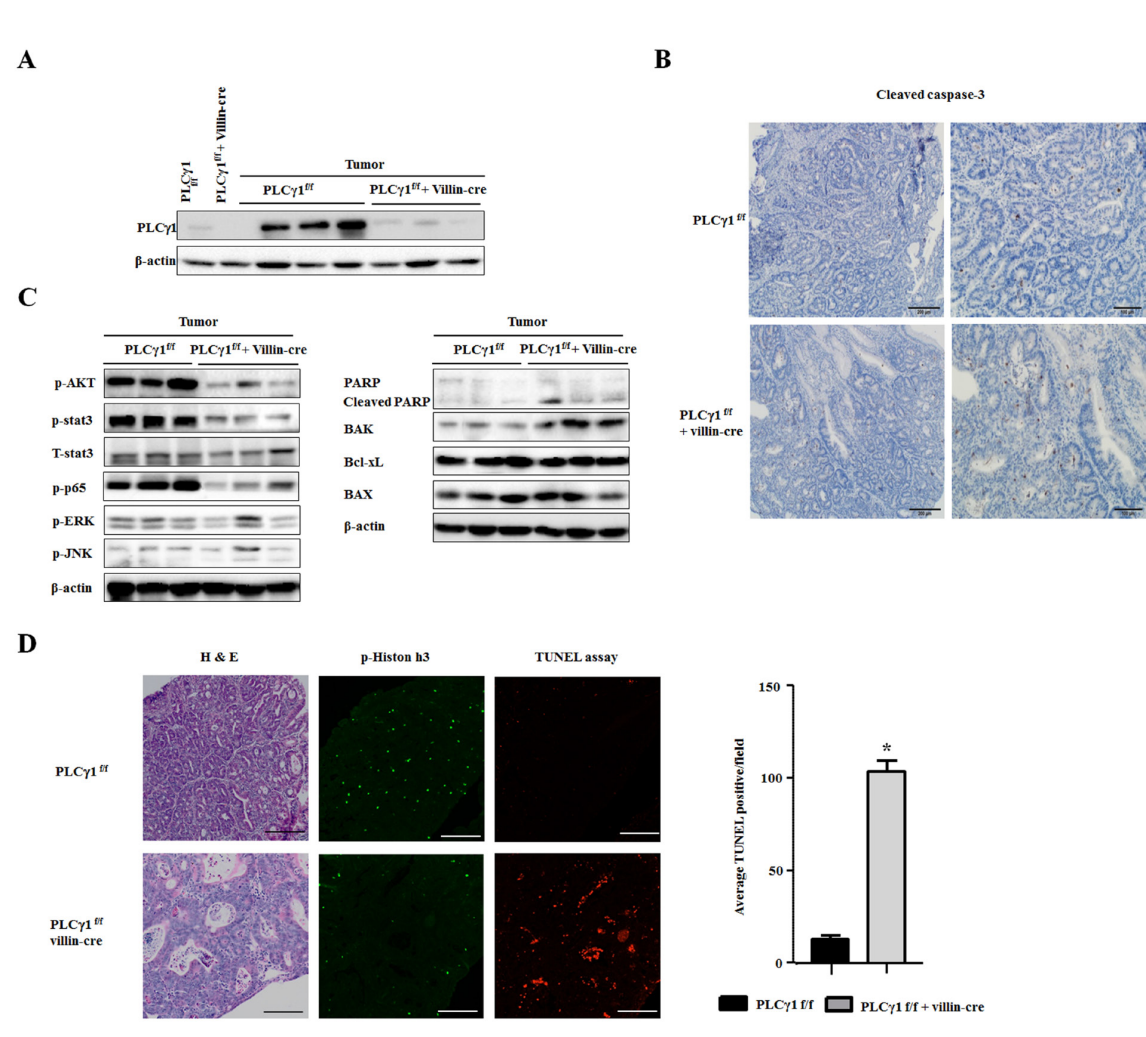
In the AOM/DSS model, deletion of PLCγ1 in IECs decreases tumor incidence by increasing apoptosis and inhibiting proliferation (**A**) Western blotting of PLCγ1 in whole-colon lysates (colon). (**B**) Colon tumor tissues were obtained from PLCγ1^f/f^ (WT) and PLCγ1 conditional knockout mice, and samples were stained for cleaved caspase-3. (**C**) Colon tumor tissues were obtained from PLCγ1^f/f^ and PLCγ1 conditional knockout mice, and samples were analyzed by western blotting (one mouse per lane) with the indicated antibodies. Data represent the means ± SEM (*n* = 3). (**D**) Colon tumor tissues were obtained from PLCγ1^f/f^ and PLCγ1 conditional knockout mice, and samples were stained for H&E staining, and p-Histone H3, and used for TUNEL assay.

Apoptosis is involved in tumor progression and maintenance. PLCγ1 regulates apoptosis enzymatically by proteolytic cleavage [20, 21]. We found that AOM/DSS induces apoptosis more strongly in PLCγ1 conditional knockout mice than in WT mice. As expected, PLCγ1 was strongly expressed in the AOM/DSS tumor model, whereas it was expressed at approximately normal levels in WT mice (Figure 2A). Numbers of cleaved caspase-3 positive cells were decreased in WT mice compared to those in PLCγ1 conditional knockout mice (Figure 2B). Analyses of apoptosis-related proteins revealed that the levels of the proapoptotic protein Bak and cleaved poly (ADP-ribose) polymerase (PARP) were significantly increased, whereas levels of phospho-AKT, phosphor-STAT3, and phospho-p65 were decreased in PLCγ1 conditional knockout mice. In contrast, Bax and Bcl-XL were expressed at similar levels in WT and PLCγ1 conditional knockout mice. The activation of c-Jun N-terminal kinase has been shown to induce apoptosis [22, 23]. However, the expression levels of mitogen-activated protein kinases were not changed (Figure 2C). We used terminal deoxynucleotidyl transferase-mediated dUTP nick- end- labeling (TUNEL) to investigate apoptotic responses in tumors from WT and PLCγ1 conditional knockout mice (Figure 2D). In contrast to WT mice, TUNEL-positive cells were detected in tumors isolated from PLCγ1 conditional knockout mice. The decrease in tumor incidence could explain the increase in apoptosis by the upregulation of Bak and cleaved PARP, and the downregulation of phospho-AKT, in tumors from PLCγ1 conditional knockout mice. The proliferation of tumor cells plays a critical role in tumor development; previous studies showed that PLCγ1 is essential for cell proliferation and differentiation [24, 25]. We tested cell proliferation in colon tumors from AOM/DSS-treated mice. We detected dividing tumor cells using p-histone H3 immunohistochemistry (IHC) and found higher proliferation rates in tumors from WT mice compared to PLCγ1 conditional knockout mice. Consistent with hematoxylin and eosin (H&E) staining, the proliferation of tumor cells was much higher in WT mice than in PLCγ1 conditional knockout mice during tumor progression and development (Figure 2D). These results suggest that the difference in tumor incidence caused by apoptosis and proliferation of tumor cells occurs through the activation of PLCγ1.

### IEC-specific deletion of PLCγ1 prevents DSS-induced inflammation

**Figure 3:**
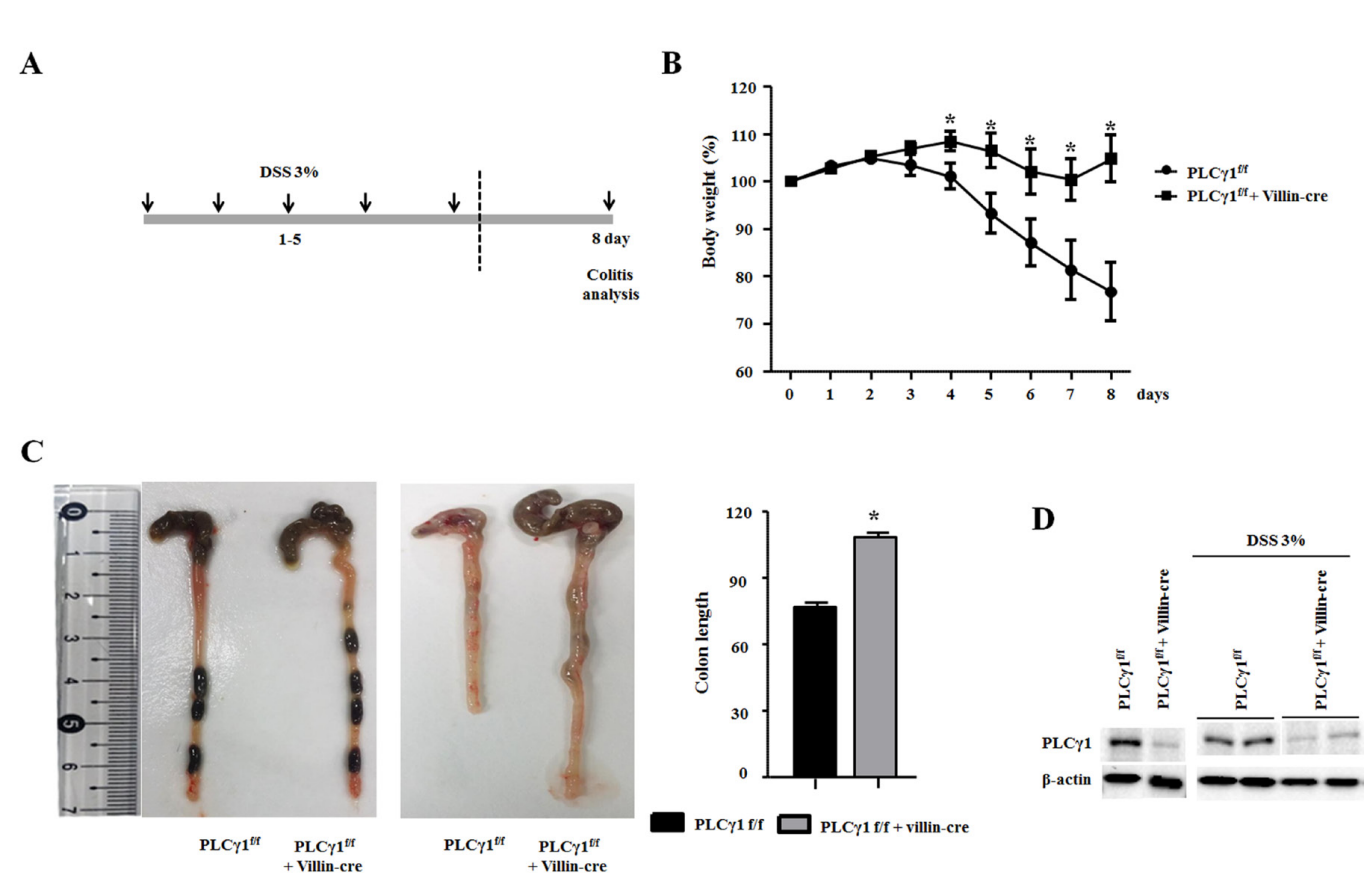
Deletion of PLCγ1 in IECs decreases DSS-induced colitis (**A**) Schematic representation of the acute colitis protocol. (**B**) DSS was administered in drinking water for five days, and body weight was recorded. Data are the means ± SEM (*n* ≥ 10), ^*^p < 0.05. (**C**) PLCγ1^f/f^ (WT) mice exhibit colon shortening after eight days of treatment with 3% DSS compared with PLCγ1 conditional knockout mice. Data represent the means ± SEM (*n* ≥ 10). ^*^p < 0.05. (**D**) Colon lysates were obtained from DSS-treated mice, and the expression of PLCγ1 by western blotting (one mouse per lane) was analyzed.

**Figure 4:**
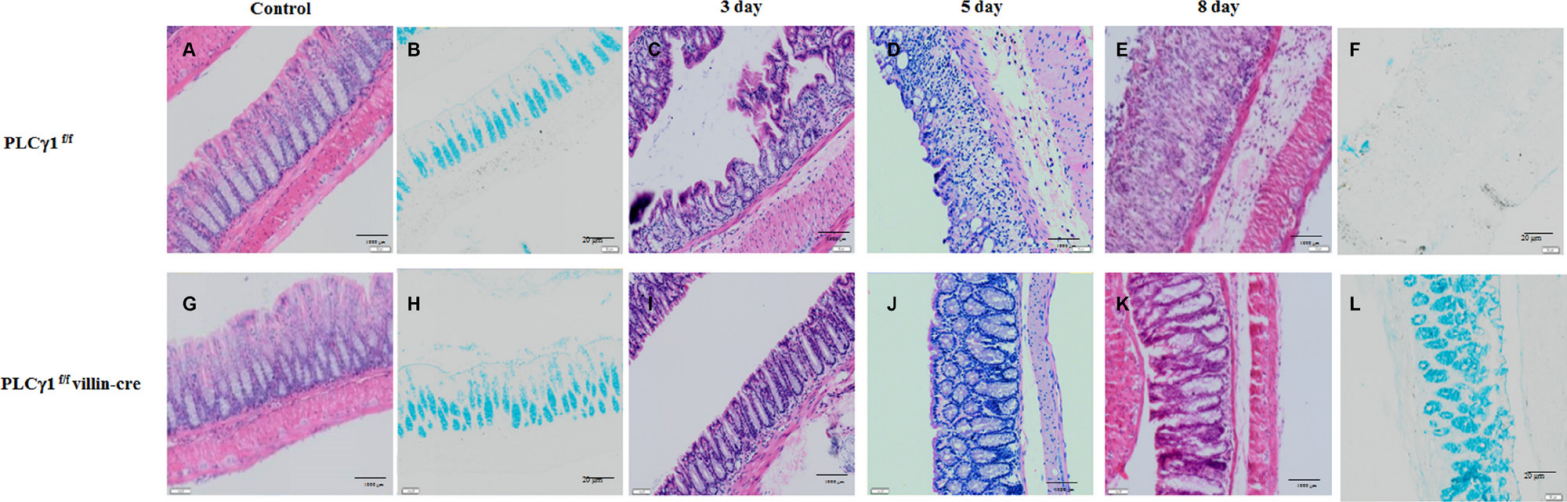
Histological changes in mice with DSS-induced colitis Decreased susceptibility of PLCγ1 conditional knockout mice treated with DSS to colitis. Colons of PLCγ1^f/f^ (WT) and PLCγ1 conditional knockout mice were analyzed by H&E staining three, five, and eight days after 3% DSS exposure, and goblet cells were analyzed by Alcian Blue staining eight days after 3% DSS exposure. (**A**, **G**) control H&E staining. (**C**, **I**) 3 days H&E staining. (**D**, **J**) 5 days H&E staining. (**E**, **K**) 8 days H&E staining. (**B**, **H**) control Alcian Blue staining. (**F**, **L**) 8 days Alcian Blue staining.

Although a recent study did not report any direct evidence for the effects of PLCγ1 inhibition on attenuated colonic inflammation, we hypothesize that PLCγ1 contributes to the inflammatory response, including via the NF-κB signaling pathway [26]. To assess whether PLCγ1 may affect colonic inflammation, we applied the DSS-induced acute colitis model in PLCγ1 conditional knockout mice and their littermate controls (Figure 3A). This method provides an indication of the mechanisms behind the initiation and/or development of AOM/DSS-induced tumors [27]. After three days of DSS administration, WT mice lost more body weight than did PLCγ1 conditional knockout mice (Figure 3B), which is an induction of the severity of DSS-induced colitis. Another indicator, DSS-induced shrinkage in colon length did not differ between PLCγ1 conditional knockout mice and WT mice (Figure 3C). The expression of PLCγ1 also did not differ between DSS-treated WT mice and PLCγ1 conditional knockout mice (Figure 3D). These results suggest that PLCγ1 stimulates inflammation and IEC damage. To confirm this hypothesis, mice were provided DSS and analyzed three, five, and eight days. At each time point, the colons of WT mice presented mild to severe colonic inflammation and a disrupted intestinal architecture, whereas the colons of PLCγ1 conditional knockout mice presented moderate to mild inflammation and a maintained intestinal architecture (Figure 4). In particular, WT mice had more infiltrating cells and fewer goblet cells compared to PLCγ1 conditional knockout mice after eight days (Figure 4). We also found that DSS-treated WT mice showed significantly more damage to IECs in the distal-to middle segments of the colon compared to PLCγ1 conditional knockout mice (data not shown).

### Deletion of PLCγ1 in IECs reduces DSS-induced inflammation

AOM and various alkylating agents induce rapid p53-dependent apoptosis of IECs by stimulating DNA damage [22]. We investigated whether AOM may further enhance apoptosis in PLCγ1 conditional knockout mice. However, AOM alone did not contribute to apoptosis via DNA damage in PLCγ1 conditional knockout mice (Supplementary Figure 2). These results suggest that the variability in tumor frequency between WT and PLCγ1 conditional knockout mice was not caused by AOM-induced DNA damage or apoptosis.

**Figure 5:**
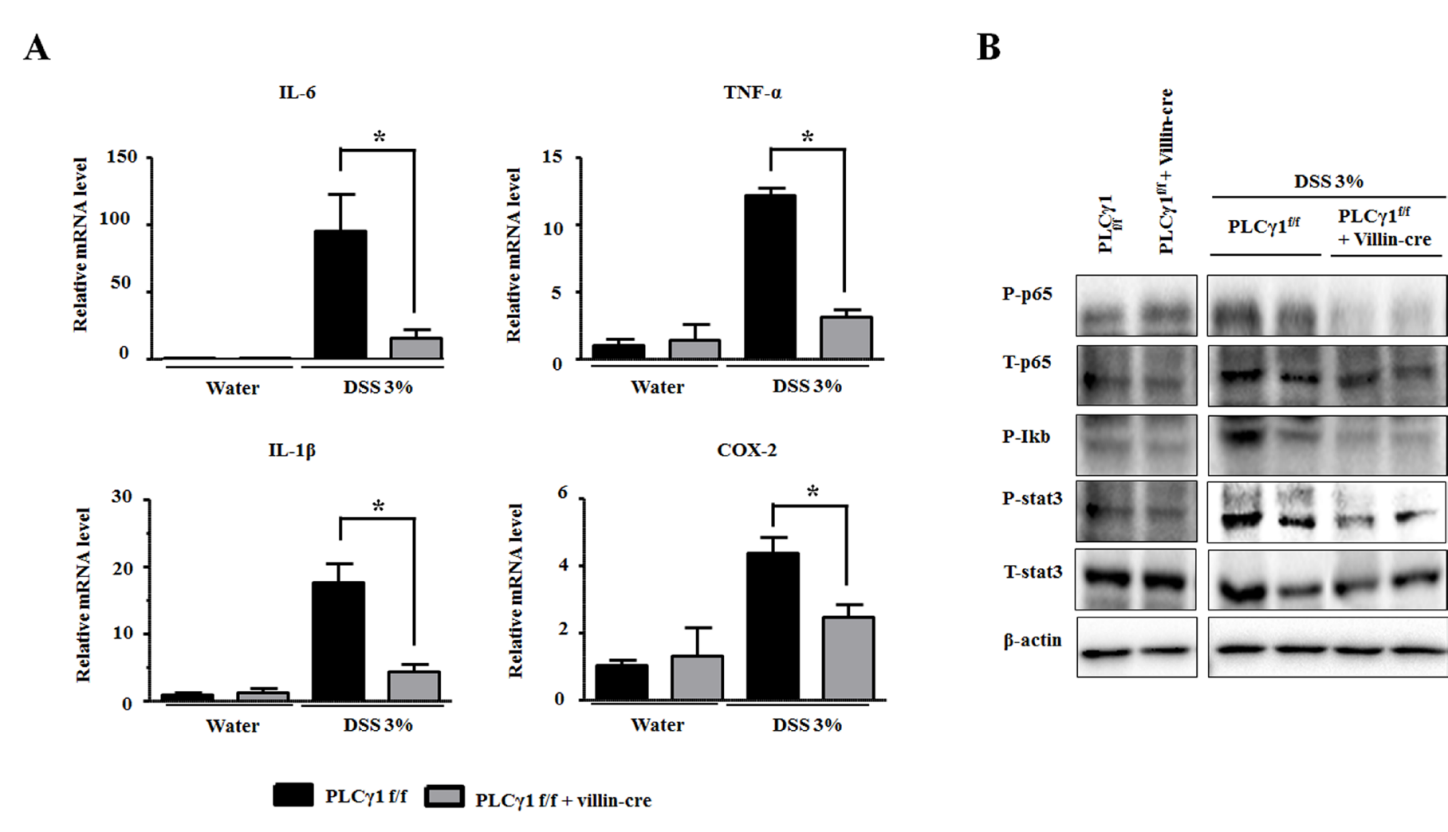
Deletion of PLCγ1 in IECs reduces DSS-induced inflammation (**A**) Relative mRNA expression levels of the indicated genes in mouse colons were analyzed by qRT-PCR. Data are the means ± SEM (*n* ≥ 10), ^*^p < 0.05. (**B**) IECs were isolated from PLCγ1^f/f^ (WT) and PLCγ1 conditional knockout mice after DSS exposure, and samples were analyzed by western blotting (one mouse per lane) with the indicated antibodies.

During colonic inflammation, cytokines and chemokines from infiltrating immune cells play crucial roles in intestinal tissues. We found that mRNA levels of cytokines, including interleukin (IL-) 6, tumor necrosis factor alpha, IL-1β, and cyclooxygenase-2, were significantly increased in colons from DSS-treated PLCγ1 conditional knockout mice compared to WT mice (Figure 5A). IL-6 levels increased more in WT mice than the other cytokines, because the PLCγ1 pathway is linked to IL-6 signaling [28, 29]. The upregulation of IL-6 enhanced inflammation via the infiltration of immune cells by paracrine and/or autocrine mechanisms [30]. In addition, we detected higher expression levels of inflammation-mediated transcription factors such as IκB, nuclear factor (NF)-κB, and STAT3; resulting in a decrease in the phosphorylation of both STAT3 and NF-κB by the activation of IκB in PLCγ1 conditional knockout mice (Figure 5B). Taken together, these results demonstrate that decreased tumor incidence in PLCγ1 conditional knockout mice is due to reduced inflammation.

### Deletion of PLCγ1 in IECs induces an intestinal barrier dysfunction after DSS treatment

**Figure 6:**
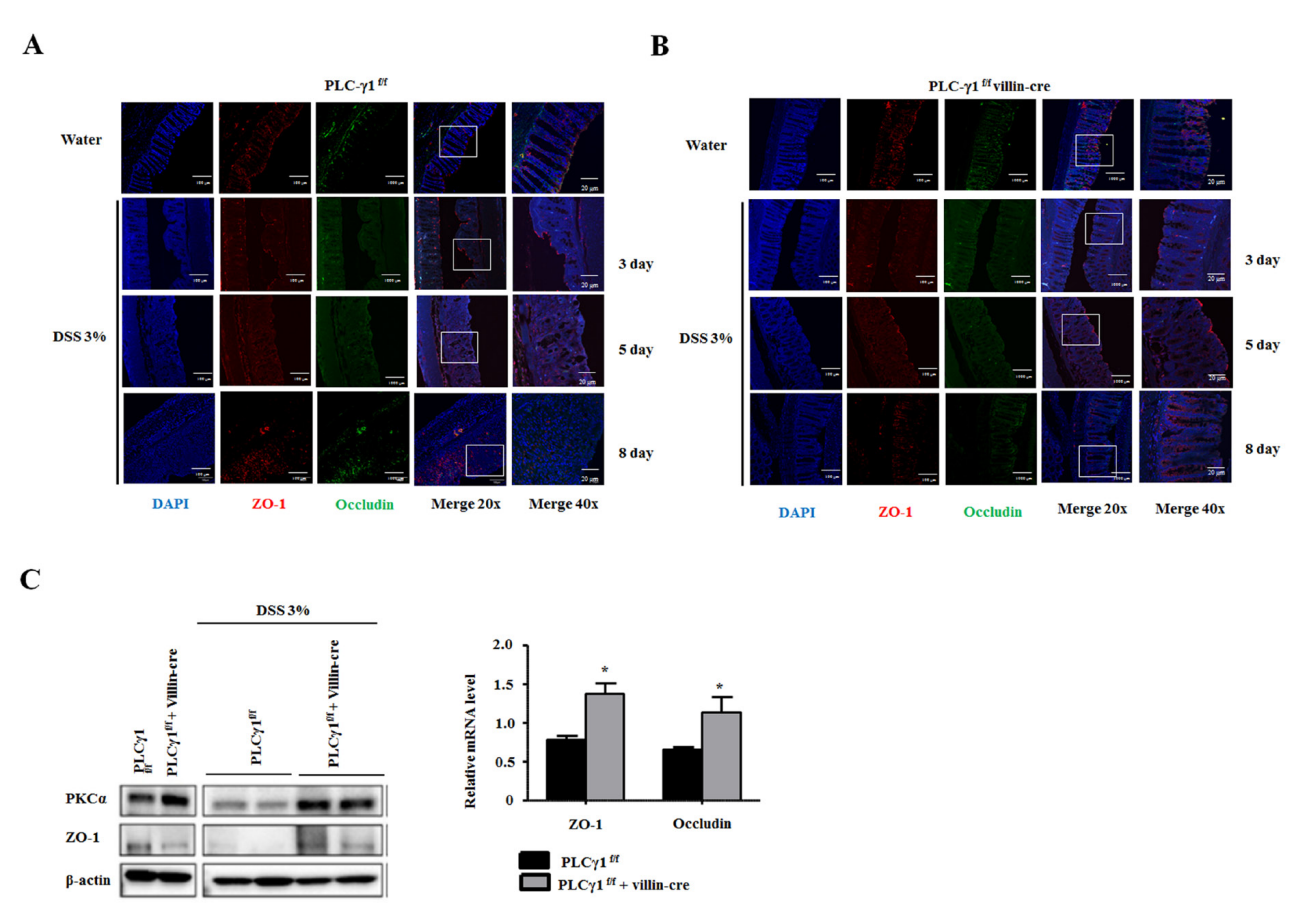
Deletion of PLCγ1 in IECs increases intestinal barrier dysfunction (**A** and **B**) DSS-induced IECs were obtained from PLCγ1^f/f^ (WT) and PLCγ1 conditional knockout mice at three, five, and eight days, and samples were stained by IHC with the indicated antibodies. (**C**) Colon lysates were analyzed by western blotting and qRT-PCR. Data are the means ± SEM (*n* ≥ 10), ^*^p < 0.05.

Disrupted epithelial barrier function has been associated with IBD and colon cancer [8, 9]. Damage to IECs leads to inflammation of the colonic mucosa as a result of intestinal barrier dysfunction in patients with ulcerative colitis [31]. Levels of IL-6 are increased in IBD patients, which modulated the expression of TJ proteins both *in vivo* and *in vitro* [32, 33]. We identified upregulated IL-6 levels in WT mice compared with PLCγ1 conditional knockout mice. We hypothesized that the DSS-induced enhancement of IEC damage and increasing inflammation in WT mice triggered intestinal barrier dysfunction. As TJs are comprised of cytoplasmic scaffolding proteins, such as ZO, occludin, claudin, and dynamic structures [34], which play pivotal roles in TJ structure and function, and recent study found that PLCγ inhibition enhanced epithelial barrier dysfunction via the regulation of TJ proteins [35], we chose to investigate the role of PLCγ1 in intestinal barrier dysfunction by analyzing the expression of TJ proteins using IHC in DSS treated colon tissues. We found that the expression levels of TJ proteins were decreased in apical lesions of the intestinal epithelium and redistributed into colonic tissues after 5 days of DSS treatment in WT mice (Figure 6A) compared to the finding that TJ proteins were maintained in PLCγ1 conditional knockout mice (Figure 6B). A difference in TJ proteins was observed after eight days of DSS exposure (Figure 6C). We also detected the expression of zonula occludens-1 (ZO-1) and protein kinase C alpha (PKCα) by western blotting. PKC activation regulates paracellular permeability via the activation of myosin light- chain kinase and the redistribution of ZO-1 [36]. We identified PKCα activation and ZO-1 downregulation in the colons of DSS-induced WT mice, and the mRNA levels of ZO-1 and occludin were significantly decreased (Figure 6C). In addition, mRNA levels and protein expression of ZO-1 and occludin were significantly decreased in colon tumors from AOM/DSS-treated WT mice Supplementary Figure 3A and 3C), but mRNA levels of IL-6 did not change (Supplementary Figure 3B). Collectively, these results indicate that a reduction in inflammation after DSS treatment in PLCγ1 conditional knockout mice is due to reduced intestinal barrier dysfunction via the inhibition of the PKC pathway and protection of TJ proteins.

## DISCUSSION

CRC is one of the most common malignant tumors [37]. Several factors have been causally associated with the development of CRC [38]. This work was designed to investigate whether the deletion of PLCγ1 in IECs could reduce colitis-associated tumor incidence (CATI) through the induction of apoptosis.

In CAC, the initial evidence for the functional relevance of PLCγ1 signaling was provided in a preclinical trial that demonstrated that many of these targets are associated with cancer initiation and progression [17]. Our data indicate that tumor progression depends on the frequency of DSS exposure, suggesting that inflammation is associated with PLCγ1 activation.

PLC hydrolyzes PIP2 into IP3 and DAG via IP3-induced calcium release, and IP3 modulated DAG activates PKC [39]. Several studies have implicated PLCγ1 as a regulator of cancer cell invasion and metastasis via multiple mechanisms. PLCγ1 is a major transducer of growth factor and cytokine signaling. Downregulation of PLCγ1 has been shown to inhibit human breast cancer cell-derived lung metastasis and progression in mice [40].

In this study, PLCγ1 conditional knockout mice and WT littermates developed colon tumors, primarily in the distal to middle segments. Only WT mice developed macroscopic tumors (>4 mm) with a higher average tumor load and tumor number than in PLCγ1 conditional knockout mice (Figure 1). Previous reports demonstrated that mutation at an important serine residue in codon 33 of β-catenin has a high oncogenic potential in AOM/DSS-induced colon adenocarcinomas [41, 42]. Therefore, we confirmed the presence of this mutation in our mice and investigated its role in the mechanism of AOM/DSS-induced colon cancer. Our results were consistent with those of previous reports [41, 42]. The mutation patterns in exon 3 of β-catenin were similar in both WT and PLCγ1 knockout mice. These results demonstrate that AOM/DSS-induces cancer using the same mechanism in both mouse groups.

AOM/DSS-treated mice consistently develop metastases; however, the absence of a control genetic mutation that can be activated by a carcinogen makes this model system of questionable utility [16]. PI(3)Kγ^–/–^ and Apc^1638N/+^ mutants have been reported to develop colonic adenocarcinomas that metastasize to the lymph nodes and liver, which are common metastatic sites of human CRCs [16]. Activated STAT3 has been shown to be increased in CRC patients [43]; its activation induces certain anti-apoptotic agents, such as Bcl-2 and Bcl-xl, which in turn increase CRC cell survival, proliferation, incursion, and lymph node metastasis [44]. Other studies have shown a correlation between STAT3 and Bak, which is probably related to the accumulation of proapoptotic molecules as well as to mediators of cell survival and growth [45]. PLCγ1 cleavage can be blocked by the overexpression of Bcl-2 and specific caspase inhibitors [20].

We examined whether PLCγ1 plays a pivotal role in IECs and whether AOM/DSS induces apoptosis in PLCγ1 conditional knockout mice. In the AOM/DSS model, the PLCγ1, Bak, and cleaved PARP levels were significantly increased in tumors isolated from WT mice, whereas cleaved caspase-3 levels were decreased; however, in PLCγ1 conditional knockout mice, phospho-AKT, phospho-STAT3, and phospho-p65 were all decreased (Figure 2). Although results from a previous report indicated that the interaction between AKT and PLCγ was involved in cell growth and migration, the extent of cross-talk between their regulatory mechanisms remain unclear [46]. However, it is well known that AKT must be phosphorylated to stimulate cell proliferation through the activated PLCγ/ PKCγ/Src/PI3K pathway in epidermal growth factor stimulated conjunctival goblet cells [40].

Recently, major proinflammatory pathways have been implicated in inflammation-associated tumor development. The carcinogenic potential of AOM is markedly increased by chronic inflammation, such as that induced by repeated cycles of DSS treatment. Furthermore, it has been reported that the IL-6/STAT3 signaling pathway plays an important role in AOM/DSS-induced murine CAC [47]. To investigate the effects of PLCγ1 on CAC tumorigenesis, particularly on the IL-6/STAT3 signaling pathway in AOM/DSS-treated mice, a deletion mutant of PLCγ1 was used in a tumorigenic model of CAC and was shown to prevent inflammation by inhibiting the phosphorylation of STAT3 on Tyr705 [48]. Here, we showed that the expression levels of STAT3 phosphorylation were higher in WT mice than PLCγ1 conditional knockout mice, leading to much higher tumor proliferation and inflammatory responses in the WT mice. As shown previously, the NF-κB-dependent tumor growth factor released by IECs could be IL-6, which plays an important role in proliferation [49] and can activates STAT3. Previous reports showed that DSS administration is well established to induce colitis, but removal of DSS leads to complete recovery of colitis to normalcy in 4–5 days [50]. IL-6 plays important roles in tumor initiation via PLCγ1–mediated inflammatory response in colitis environment, and also after recovered colitis by removal of DSS, crosstalk between stat-3 and PLCγ1 is involved in tumor progression and development through apoptosis and proliferation. This result could elucidate the role of PLCγ1 in the suppression of the IL-6/STAT3 pathway; and provide further insight into the critical mechanisms involved (Figures 2C and 5).

The cellular localization of most PLCs is primarily cytosolic in the resting state; following receptor activation, PLCs are transiently recruited to the cell membrane. PLC-dependent pathways are postulated to be involved in the regulation of TJs [51]. In general, TJ formation is essential for early embryonic development, and PLCs may plays crucial roles in such developmental processes [52], a hypothesis supported by the developmental defects observed in various knockout mice lacking different PLC isozymes. PLCγ is known to contain SH3 domains that bind proline-rich motifs on target proteins; interestingly, ZO proteins contain proline-rich sequences that could bind and localize PLCγ to TJs [53]. Although no reports have described the colocalization of PLCγ and TJs, PLC isozymes have been reported to colocalize with cortical actin filaments. Recently, PLCγ1 was detected in human T cells as a phosphoprotein [54]. CD3 activation of T cells was shown to cause tyrosine phosphorylation of PLCγ1, which is associated with a marked increase in PLC activity that accounts for the activation and translocation of PKCα and results in low expression levels of ZO-1 and claudin-2, thereby opening TJs in the epithelium [55]. Our data indicate that the reduction in inflammation after DSS treatment of PLCγ1 conditional knockout mice is due to reduced intestinal barrier dysfunction via PKC pathway blockade and protection of TJ proteins via PKCα (Figure 6).

Our data show that when PLCγ1 is lacking in IECs, cancer incidence is reduced. It is possible that tumor incidence depends on the frequency of DSS exposure and is associated with a PLCγ1-stimulated inflammatory response that affects tumor expression. Our study suggests that PLCγ1 signaling may be a good target for therapies to reduce CATI. Indeed, PLCγ1 is known to play important normal physiological roles, such as cell survival; and proliferation, as well as roles in cancer metastasis, such as angiogenesis and vascular permeability. The link between PLCγ1 activity and the IL-6/STAT3 signaling pathway could account for the transcription of many important cancer-related genes. Although we have shown that the knockdown of PLCγ1 induces apoptosis in the AOM-DSS model by mechanisms involving the downregulation of the IL-6/STAT3 signaling pathway, we aimed to demonstrate that PLCγ1 may be an effective treatment for CRC by blocking the IL-6/STAT3 signaling. Studies of the function of PLCγ1 are needed to elucidate potential novel targets for therapeutic intervention and provide new insights into the extent of PLCγ1 participation in CATI.

## MATERIALS AND METHODS

### Animals and induction of colitis and CAC

PLCγ1^f/f^ (WT) and PLCγ1 conditional knock-out mice were generated with Villin-Cre mice [56]. The experiments were performed using 6–8 weeks old age-matched mice in a C57BL/6 background. Their floxed littermates were used in all experiments as controls. Mice housing conditions and experimental protocols were approved by the Institutional Animal Care and Use Committee at Ulsan National Institute of Science and Technology. CAC was induced as described (Greten *et al.*, 2004). Briefly, on day 1, mice were IP injected with 10 mg/kg AOM (Sigma- Aldrich) and maintained on regular diet and water for 5 days. After 5 days, mice received water with 2% (unless stated otherwise) DSS (dextran sulfate sodium, MP Biomedicals, molecular weight 35,000–50,000) for 5 days (DSS one cycle), followed by regular water for 14 days, and this protocol were performed during two further DSS treatment cycles (DSS three cycles). Mice were sacrificed at 100 days (DSS three cycles) and 120 days (DSS one cycle) after AOM injection, respectively. For acute colitis and inflammation studies, mice were administered 3% DSS for 5 days, followed by regular water for 3 days and sacrificed at the indicated time points. Body weights were recorded during DSS treatment. Colon length was analyzed after removal from mice and flushed with cold phosphate-buffered saline (PBS), opened longitudinally, following which tumor number and size measurements were recorded. One half of the distal colon was used for Western blot and quantitative polymerase chain reaction (qPCR) analysis. The other half the colon tissues were fixed as ‘‘swiss-rolls’’ in 10% formalin solution (Sigma, #HT-501128) at room temperature overnight, and paraffin embedded.

### Histological analysis

Paraffin-embedded colons were cut into 5 μm thick sections, and serial sections were stained with H&E and Alcian Blue (for goblet cells). Using Scion Image for Windows (Zeiss), the colitis-severity and width of each tumor was measured and recorded.

### Immunoblotting

Colon tissues were rinsed with ice-cold PBS and lysed using the radio-immunoprecipitation assay (RIPA) lysis buffer (Millipore Corporation, Billerica, MA, USA). Tissue lysates were centrifuged at 14,000 × g for 30 min. The supernatant was collected, and protein was measured using the BCA Protein Assay Kit. The proteins were separated by sodium dodecyl sulfate polyacrylamide gel electrophoresis (SDS–PAGE) gel and were electrophoretically transferred to polyvinylidene fluoride (PVDF) membrane (Millipore Corporation, Billerica, MA, USA). The membranes were blocked in 5% skimmed milk in Thermo Scientific SuperBlock (TBS) containing 0.05% Tween-20 (TBST) buffer for 1 h, and then incubated with primary antibodies overnight at 4°C. After washing in TBS-T buffer, the membranes were incubated with secondary antibodies for 1 h at room temperature. Protein bands were detected with Immobilon Western substrate (Millipore Corporation, Billerica, USA) and analyzed with the ChemiDoc Touch Imaging System (Bio-Rad, Hercules, CA, USA).

### Immunohistochemistry

Sections of paraffin-embedded colon tissues were dewaxed in xylene, rehydrated, and subjected to antigen retrieval in 10 mM citrate buffer. Sections were blocked for 30 min in 1% bovine serum albumin, 0.02% Triton X-100, and 5% normal goat serum (NGS). Serial sections were incubated with rabbit polyclonal ZO-1 antibody (1:200 dilution) and occludin antibody (1:200 dilution), followed by Cy5-conjugated goat anti-rabbit secondary antibody (Jackson ImmunoResearch Laboratories, Inc., West Grove, PA, USA). The sections were mounted using Vectashield with 4′,6-diamidino-2-phenylindole (DAPI) (Vector Laboratories, Burlingame, CA, USA). Images from serial sections were acquired using an Axioskop inverted microscope with an AxioVision camera and software (Zeiss, Thornwood, NY, USA).

### RNA extraction and quantitative reverse transcription polymerase chain reaction

Total RNA was extracted from distal colon segments or from colon tumors using TRIzol (Invitrogen, Carlsbad, CA, USA) and QIAGEN RNAeasy isolation kits (QIAGEN, Valencia, CA, USA). qRT-PCR was performed in triplicate using 4 μL of 1/12 diluted cDNA and SYBR green (Bio-Rad #1708886) in 20 μL total volume on a Roche 480 (Roche, Basel, Switzerland). The mRNA level was calculated by the cycle threshold (Ct) value and was normalized by glyceraldehyde 3-phosphate dehydrogenase (GAPDH).

### Detection of tumor mutations

Tumor tissues were obtained by laser capture microdissection (LCM) from paraffin-embedded colons and were digested with proteinase K. Genomic DNA was purified by standard phenol chloroform extraction. Exon 3 of the β-catenin gene was amplified by PCR using the following specific primers: 5′-GCTGACCTGATGGAGTTGGA-3′ and 5′- GCTACTTGCTCTTGCGTGAA-3′ (amplicon size = 227 bp). PCR products were purified and sequenced using forward and reverse primers. Mutations were detected by observing individual chromatograms.

### Statistical analysis

Data are expressed as mean ± standard error of the mean (SEM). Differences were analyzed by Student’s *t* test. Values of *p* < 0.05 were considered significant.

## SUPPLEMENTARY MATERIALS FIGURES


