# Supplementary material for: Phospholipase Cγ1 links inflammation and tumorigenesis in colitis-associated cancer
Source: Oncotarget. 2017 Dec 19;9(5):5752–63. doi: 10.18632/oncotarget.23430 (PMC5814171; doi:10.18632/oncotarget.23430)
Supplement: Supplementary file 1 [file oncotarget-09-5752-s001.pdf]

Phospholipase Cγ1 links inflammation and tumorigenesis in colitis-associated cancer

SUPPLEMENTARY MATERIALS

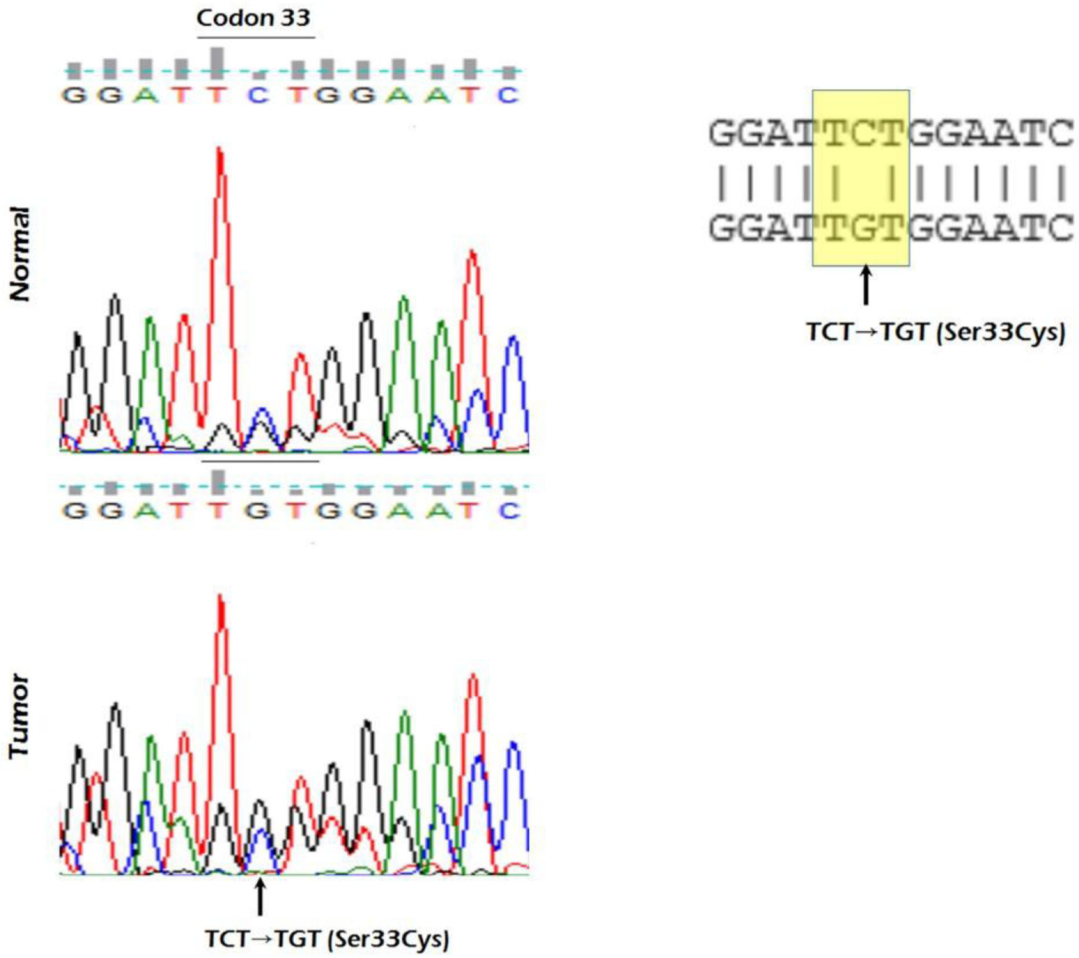

Supplementary Figure 1: Analysis of tumors harboring β-Catenin mutations. Mutations within exon 3 of the β-Catenin gene. DNA was eluted by laser capture microdissected tumor cells. Exon 3 that codes for the GSK-3β phosphorylation site (codon 33) was mutated from serine to cysteine.

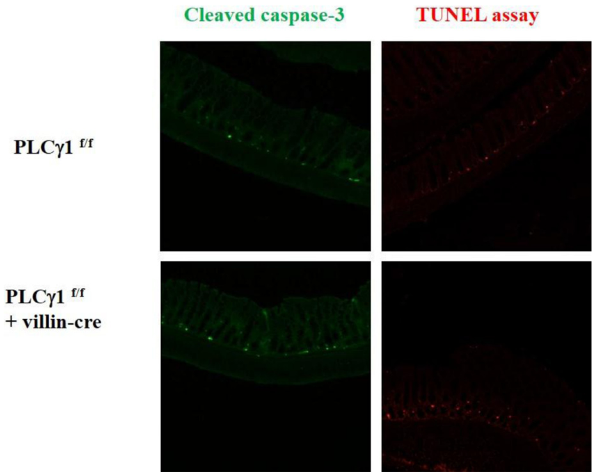

Supplementary Figure 2: Deletion of PLCγ1 in IEC does not affect AOM-induced DNA damage. PLCγ1$^{f/f}$ (WT) and PLCγ1 conditional knockout mice were intraperitoneally injected with AOM and scarified after 7 h, following which colon tissue sections were stained with cleaved caspase-3 and terminal deoxynucleotidyl transferase-mediated dUTP nick-end-labeling (TUNEL) assay for analyzing apoptosis.

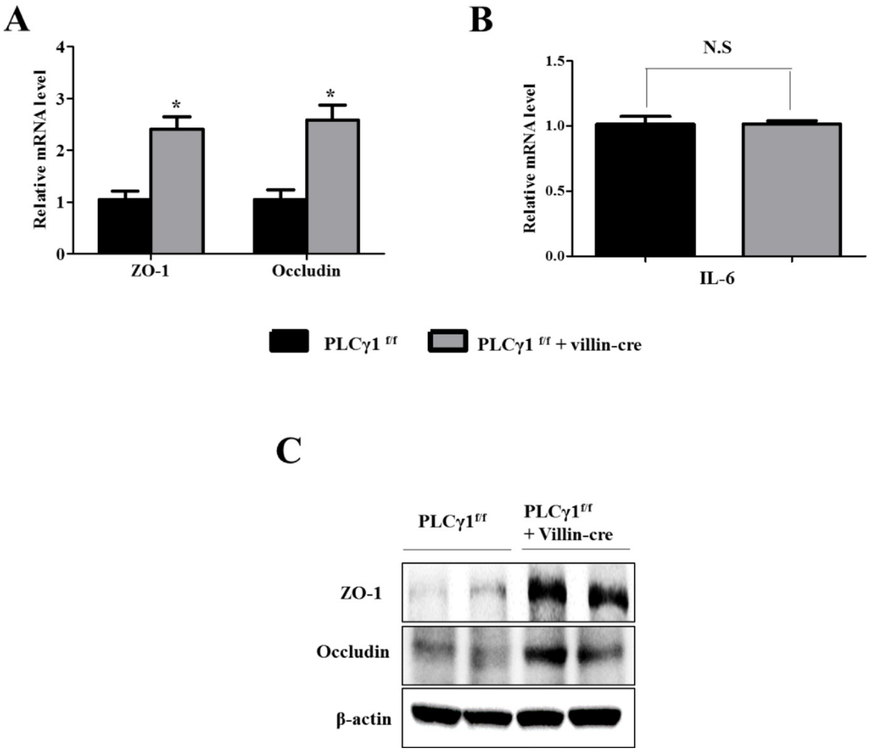

Supplementary Figure 3: The mRNA levels of ZO-1 and occludin decrease in AOM/DSS-induced tumor, but IL-6 does not change. Colon lysates were analyzed by qRT-PCR (**A** and **B**) and western blotting (**C**). Data are the means ± SEM ($n \geq 10$), $^{*}p < 0.05$.
